# Sex Steroid Hormones Regulate Leptin Transcript Accumulation and Protein Secretion in 3T3-L1 Cells

**DOI:** 10.1038/s41598-017-07473-5

**Published:** 2017-08-15

**Authors:** Mónica Z. Jenks, Heather E. Fairfield, Erik C. Johnson, Ron F. Morrison, Gloria K. Muday

**Affiliations:** 1 Department of Biology and Center for Molecular Signaling, Wake Forest University, Winston Salem, North Carolina USA; 2 Department of Nutrition, University of North Carolina at Greensboro, Greensboro, North Carolina USA

## Abstract

Leptin is an adipokine produced by fat cells that regulates food consumption and metabolic activity. Sexual dimorphism in leptin and fat stores have been observed in humans and rodents with females having more leptin and greater levels of subcutaneous fat than males. One potential mechanism leading to this dimorphism is steroid hormone regulated synthesis of transcripts encoding leptin. Identification of direct regulatory mechanisms is difficult in animals or primary adipocytes due to these intertwined dimorphisms. We used well-characterized 3T3-L1 murine adipocytes to demonstrate that dihydrotestosterone (DHT) reduced *Leptin (Lep)* transcript abundance and cytosolic and secreted leptin protein. The magnitude of this effect was greatest on secreted leptin, which was decreased by DHT to 30% of the control. In contrast, 17β-estradiol significantly increased the abundance of transcripts encoding leptin and increased secreted leptin to 230% of the control. Treatment with estrogen and androgen receptor antagonists had opposite effects on *Lep* transcript abundance to steroid treatments, indicating that these transcriptional effects are mediated through the canonical steroid hormone signaling pathways. These results indicate that short-term treatments with steroid hormones are sufficient to alter both *Lep* transcript accumulation and leptin protein secretion, and may play a role in the sexual dimorphism of this adipokine.

## Introduction

The rates of obesity are increasing worldwide, with recent estimates suggesting that more than a third of adults in the United States are obese^1, 2^. Increases in *Diabetes mellitus*, heart disease, and hypertension parallel the obesity trends^3^. Insight into the hormonal and metabolic changes that accompany obesity is needed to explain the linkage to these disease states. There are large differences in the metabolic complications associated with obesity between males and females^4–6^. The reduced prevalence of obesity-related metabolic disorders in women under the age of 50, and the increases of these metabolic disorders after menopause suggests that sex steroid hormones may play central roles in these responses^7, 8^.

The identification of adipokines, which are signaling molecules secreted by adipose tissues^9^, has provided insight into how fat stores signal to coordinate metabolism^10, 11^. Leptin, an adipokine that represses food consumption, has been extensively studied in both mice and humans^12–15^, where mutations in the Leptin gene (*Lep*) result in obesity^12, 15, 16^. Leptin is secreted by fat cells and circulates in the plasma of mammals inducing a variety of metabolic, neuronal, and inflammatory signals^17^. Plasma leptin levels in humans are proportional to body mass index (BMI) and total body fat, suggesting the model that when energy stores are plentiful, leptin production may be increased to signal satiety^16^. When individuals reduce their body weight and fat stores through increased exercise and/or dietary modifications, leptin levels also decrease^18, 19^. Leptin signals satiety by binding to its receptors and increasing the synthesis of neuropeptides that reduce the activity of brain circuits that drive food consumption^20, 21^. In a recent review on the 20 years of research on leptin, Friedman lists understanding of the mechanisms that control leptin synthesis as an important future goal^17^.

Leptin levels and body weight maintenance are sexually dimorphic in humans, nonhuman primates, and rodents^5, 8, 22–24^. The average mass of fat stores is higher in women than men, and distribution of fat stores differ with females having greater subcutaneous fat and less visceral fat than males^22^. Women also have approximately 40% higher leptin levels than men even after compensating for BMI, adiposity, and age^24^. The possibility that these leptin levels are regulated by sex steroid hormones is suggested by examination of the changes in leptin across sexual maturity. At puberty, leptin levels increase in females, but decrease in males^25^. Additionally, serum leptin concentrations are lower in post-menopausal women than in younger women^26^. A human cross-sectional study in young men and women showed that leptin levels are positively correlated with estrogen concentration and negatively correlated with testosterone concentration^27^. Studies in humans undergoing steroid hormone treatments as part of gender reassignment^28^, and in rodents with altered androgen receptor signaling^29^ or surgery to remove ovaries or testes^23^ yielded differences in both fat stores and leptin levels.

Prior studies are consistent with regulation of leptin synthesis by sex steroid hormone in primary cell cultures and animals^30–32^. Working with tissues and primary cell cultures can be challenging due to limited access to samples and high variability between individuals, as well as developmental changes resulting from hormone treatments. This study used a well-characterized murine adipocyte cell line (3T3-L1)^33–39^ to strengthen our understanding of the relationship between steroid hormones and transcriptional controls of leptin synthesis and to complement these previous studies. This cell line has been shown to express transcripts encoding both androgen and estrogen receptors, suggesting it may be able to respond to these two hormones^40–42^. We found that DHT treatments resulted in a significant decrease in *Lep* transcript abundance, and in cellular and secreted leptin protein levels. In contrast, estradiol treatment resulted in significant increases of *Lep* transcript abundance and in secreted leptin protein. These transcript abundance changes were reversed by androgen and estrogen receptor antagonists. Our results are consistent with the sexual dimorphism observed in humans and rodents due to an inhibitory effect of DHT in males on *Lep* transcript abundance and a stimulatory role of estradiol in females, resulting in lower circulating leptin levels in men compared to women.

## Materials and Methods

MediaTech Dulbecco’s Modified Eagle’s Medium (25 mM glucose), calf serum, Halt Protease Inhibitor Cocktail, Pierce Phosphatase Inhibitor Mini Tablets, and Pierce Micro BCA Protein Assay kit, Precise Tris-Glycine 8–16% gradient gels, Thermo Scientific PVDF transfer membrane, and Millipore Immobilon Chemiluminescent HRP substrate were purchased from Fisher Scientific Company (Pittsburgh, PA). DNase I was purchased from Promega (Madison, WI). Fetal bovine serum was purchased from Atlanta Biologicals, Inc (Lawrenceville, GA). Purified recombinant mouse leptin was purchased from the Harbor-UCLA Research and Education Institute. Amicon Ultra centrifugal filter units and mouse leptin ELISA kits (EZML-82K, sensitivity = 0.2 ng/ml) were purchased from Millipore (Billerica, MA). Oil Red O, 17β-estradiol, dihydrotestosterone, bovine insulin, dexamethasone, and methyl-isobutyl-xanthine (MIX), anti-leptin polyclonal antibody produced in rabbit (L3410), and sodium deoxycholate were purchased from Sigma-Aldrich (St. Louis, MO). The beta-actin monoclonal antibody produced in rabbit (8457 S) and HRP-linked secondary antibody (7074P2) were purchased from Cell Signaling Technology (Danvers, MA). The RNeasy RNA extraction kits were purchased from QIAGEN (Valencia, CA). SuperScript III First-Strand Synthesis System for RT-PCR was purchased from Invitrogen (Carlsbad, CA). SYBR Green dye was purchased from Applied Biosystems (Carlsbad, CA). Leptin primers were designed with Primer3Plus Software. Leptin, 18S [28], and estrogen receptors alpha and beta [29] primers were synthesized by Integrated DNA Technologies (Supplemental Table 1).

### Cell Culture

Murine 3T3-L1 preadipocytes were obtained from Howard Green at Harvard Medical School, who originally isolated this cell line^43, 44^ and this cell line has been used extensively by these authors^33–39^. This cell line is unique in its ability to be differentiated to adipocytes, which leads to lipid droplet accumulation that can be visualized by Oil Red O staining, as shown in Supplemental Fig. 1. Differentiation is also confirmed with well-established gene expression profiles^37^. Cells are always used at less than 25 passages. 3T3-L1 cells were cultured as described previously^33^. Briefly, cells were incubated at 37 °C with 5% CO_2_ in Dulbecco’s modified Eagle’s medium (DMEM) supplemented with 10% calf serum. At two days post-confluence, cells were treated with a differentiation cocktail (MDI) containing 0.5 mM methyl isobutyl-xanthine, 1 µM dexamethasone, 1.7 µM insulin in media supplemented with 10% fetal bovine serum (FBS). Complete differentiation (>90% of cells) 8 days after MDI treatment was verified either through visualization of lipid droplets in the cytoplasm by light microscopy or staining with Oil Red O, as described previously^37^. For all experiments separate biological replicates are reported with each sample from a separate set of cultured cells, with all experiments within the manuscript using three different starting batches of cells.

For Oil Red O staining, cells were incubated in 10% formalin (in PBS) for 5 minutes. Cells were incubated in fresh formalin for 1 hour. Formalin was removed and cells were washed with 60% isopropanol. Once the plate was completely dry, 5 ml of Oil Red O was added and allowed to incubate for 10 minutes. The dye was removed and cells were washed with water 4 times. Images were acquired with the inverted Zeiss Axioplan microscope, Hamamatsu Orca-ER camera, and Volocity software. A representative image illustrated the appearance of lipid droplets in differentiated cells in the absence or presence of estradiol or dihydrotestosterone (Supplemental Fig. 1). Images were adjusted in Adobe Photoshop to optimize color balance and contrast.

### Steroids and receptor antagonist treatment

Steroid hormone treatments were performed at eight days after differentiation in cells that were serum starved for 6 hours prior to treatment. As phenol red has been reported to possibly mimic estrogens, experiments were performed in cells that were transitioned to phenol red-free DMEM at day 6. On day 8, cells were serum deprived for 6 hours and then treated for 24 hours with doses of 17β-estradiol and DHT ranging from 0.1–100 nM in serum-free media. Inhibitors (1 µM fulvestrant, which blocks both α and β estrogen receptors, and 200 nM flutamide^45–47^, which blocks androgen receptor signaling) were added to cells for 2 hours prior to hormone treatments. Each transcript and protein value represents separate biological samples with the replicates noted for each data representation.

### RNA extraction, cDNA synthesis and quantitative real time PCR (qRT-PCR)

Cells were detached from 100 mm tissue culture plates with 650 µl RLT buffer. RNA was harvested from cells using the RNeasy RNA extraction kit using the manufacturer’s recommendations (QIAGEN). RNA was eluted from the column with water and quantified with a Nanodrop spectrophotometer (Thermo-Fisher) and treated with DNase I.

cDNA synthesis was performed using a first strand synthesis kit with SuperScript II reverse transcriptase (Life Technologies) with at least 2 µg of RNA, followed by treatment with RNase H to remove the RNA precursors. The cDNA was then used for quantitative real-time PCR with SYBR Green (Applied Biosystems). Reactions were run in 96-well plates on the 7500 Fast Real-Time PCR System (Applied Biosystems) at 50 °C for 2 minutes, 95 °C for 10 minutes, and then for 40 cycles of 95° for 15 seconds, 60 °C for 1 minute, 95 °C for 30 seconds, and 60 °C for 15 seconds. Three technical replicates were run for each reaction and the average of three technical replicate is counted as a single biological replicate. Abundance of each transcript was normalized to an 18S rRNA transcript control. Because the efficiency of each primer set varies, graphs of Ct over a range of DNA concentrations were constructed for each primer set and the equation of these lines were used to calculate the levels of each transcript. The fold change values are reported relative to untreated samples and represent at least 3 biological replicates from at least three different cultures.

### Western Blots

Cells were harvested with a protein extraction buffer containing 150 mM sodium chloride, 50 mM Tris (pH 7.4), 1% NP-40, 0.5% sodium deoxycholate, 0.1% SDS, 1X Halt protease inhibitor cocktail, and Pierce Phosphatase Inhibitor Mini Tablets. Supernatants were also collected and proteins were concentrated using Amicon Ultra 10k centrifugal filter units. All samples were spun at 14,000 × *g* for 15 minutes and protein concentrations were determined using a Micro BCA assay. Equal amounts of protein from each sample were run on 8–16% gradient SDS-PAGE gels and transferred to PVDF membranes. Actin blots were run as an additional loading control for both cell extracts and supernatants, as this protein has been shown to accumulate in samples enriched in extracellular, secreted vesicles^48^. Blots were blocked in Tris-buffered saline solution containing 5% non-fat milk and 0.05% Tween 20 for at least 2 hours and then were incubated with primary antibodies overnight at 4 °C. The leptin antibody was diluted 1:500, and the β-actin antibody was diluted 1:100. The HRP-conjugated secondary antibody was used at a 1:10,000 dilution. Bands were visualized with a chemiluminescent HRP substrate and an Amersham Imager 600 and quantified with the ImageQuant TL software (GE). Purified mouse leptin was used as a positive control on blots to identify the size of the leptin band and to verify the specificity of antibody recognition.

### Statistical Analyses

Two-sample, independent Student’s t-tests were performed in Excel for hormone dose response treatments on *Lep* transcript abundance and leptin protein accumulation and secretion. In the experiments using receptor antagonists, flutamide and fulvestrant, and steroid hormones, there were more than 2 treatment types and the data was non-parametric, therefore a Kruskal-Wallis test was performed using GraphPad Prism 7.0 software to determine if there were significant differences within these data sets. To determine which treatments were significant, pairwise comparisons were made using two-tailed Mann-Whitney tests. P-values for these tests are reported in the results section. All data values were reported as mean ± standard error (SE).

## Results

### Dihydrotestosterone treatment significantly decreased abundance of transcripts encoding leptin

**Figure 1:**
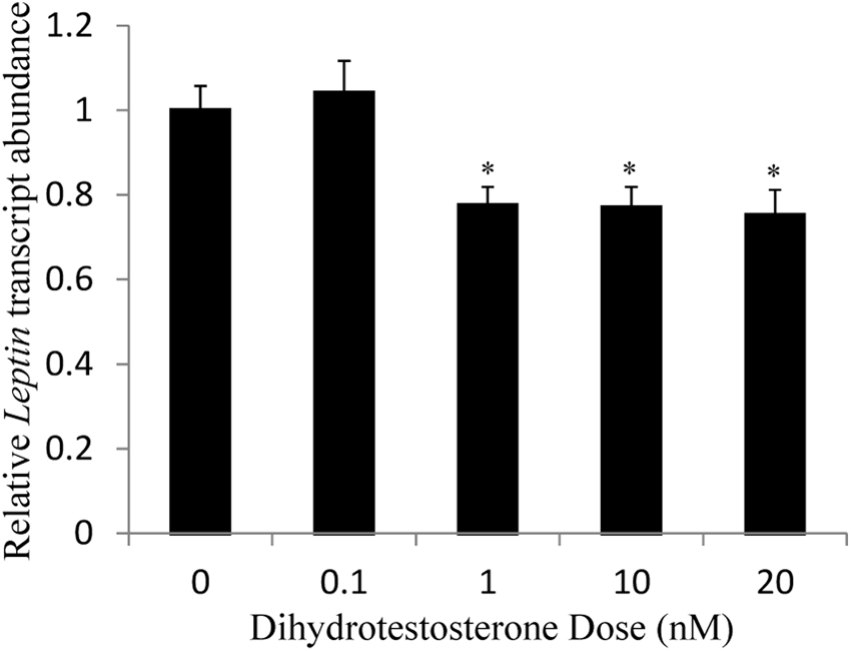
*Lep* transcript abundance decreased at elevated DHT levels. 3T3-L1 cells were treated with the indicated dose of DHT for 24 hours. Leptin transcripts were measured by qRT-PCR and normalized to an 18 S rRNA control. The transcript abundance relative to untreated controls is reported as mean ± S.E.M with n ≥ 8 biological replicates; *Denotes p-value ≤ 0.05 when compared to control with 2-sample, independent Student’s t-tests.

We tested the hypothesis that leptin levels are lower in males than females due to an inhibitory effect of DHT on transcription of the gene encoding leptin. RNA was isolated from 3T3-L1 cells treated with DHT for 24 hours at concentrations ranging from 0.1 to 20 nM. The levels of *Lep* transcripts relative to an 18S ribosomal subunit control were determined by qRT-PCR and values were reported relative to an untreated control with n ≥ 8 biological replicates (individual aliquots of cultured cells from at least 3 batches of cells). For each sample, three technical qRT-PCR replicates are averaged to provide one biological replicate. A statistically significant DHT-dependent decrease in *Lep* transcripts was detected at doses of 1, 10, and 20 nM (p-values ≤ 0.004) (Fig. 1). This DHT dose is consistent with physiological concentrations observed in young adult men (approximately 1 nM)^49^. The maximum response observed was a 25% reduction at 20 nM DHT.

### Androgen receptor antagonists prevent the DHT effect on *Lep* transcripts

**Figure 2:**
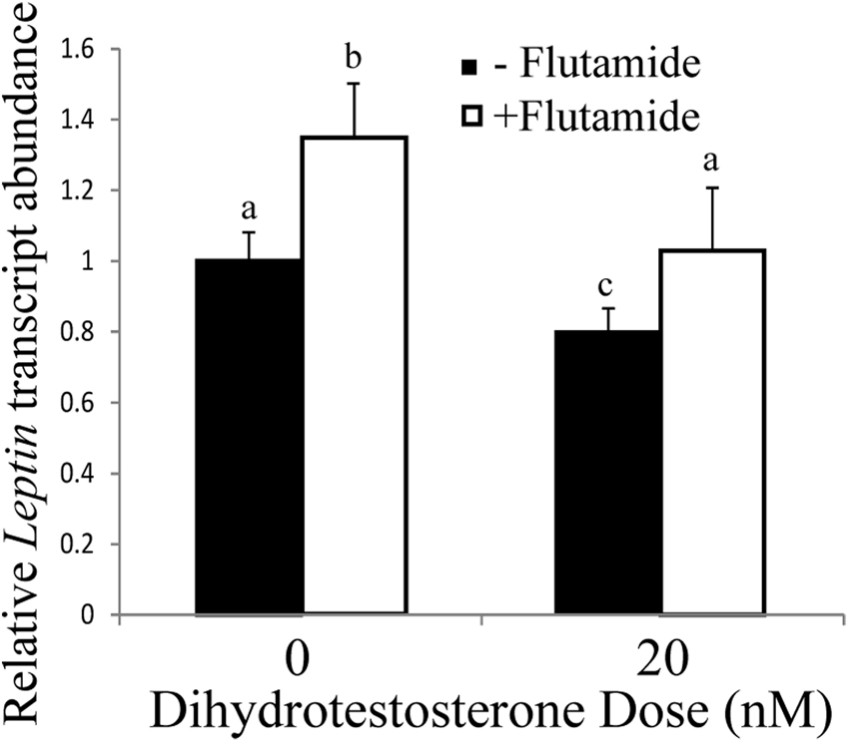
*Lep* transcript abundance decreased with 20 nM DHT and increased with 200 nM flutamide treatment. Leptin transcripts were measured by qRT-PCR and normalized to an 18 S rRNA control. The transcript abundance relative to untreated controls is reported as mean ± S.E.M with n ≥ 6 biological replicates. Means with different letters are statistically different using two-tailed Mann-Whitney tests.

To demonstrate that the observed decrease in *Lep* transcript levels after DHT treatment was mediated by androgen receptor signaling, flutamide, a competitive inhibitor of DHT binding to its receptor, was used to block androgen receptors in the adipocytes. Cells were pretreated with 200 nM flutamide for two hours before addition of 20 nM DHT (or mock treatment) for an incubation of 24 hours. *Lep* transcript abundance was measured after treatments with qRT-PCR. A Kruskal-Wallis test examined the difference in transcript abundance between the different treatments χ^2^(2) = 7.28, *p* = 0.06. The statistical significance between treatments was examined in pairwise Mann-Whitney comparisons. An increase in leptin transcript abundance was observed with flutamide alone (*p* = 0.07), consistent with an inhibition of androgen-mediated repression of leptin synthesis by its ability to block binding of endogenous DHT^50, 51^ in these cells and reduce expression of the androgen receptor^52^ (Fig. 2). DHT also decreased *Lep* transcript abundance (*p* = 0.07). The largest effect was the 2-fold difference between DHT and flutamide treatment (*p* = 0.01). In contrast, the combined treatments with flutamide and DHT were identical to the untreated control (*p* = 1.0) (Fig. 2). The restoration of leptin transcript levels in the presence of both flutamide and DHT to untreated controls is consistent with the action of flutamide as a competitive inhibitor of this DHT binding. DHT reduces the inhibition of receptor activity by flutamide, reducing transcript levels, while flutamide prevents the inhibitory effect of DHT on leptin transcripts by blocking its binding to the receptor. These experiments were performed with cells that were in serum-free media for 30 hours, with the goal of eliminating exogenous steroid hormones and reducing endogenous levels. Our attempts to quantify the steroid hormone levels after these treatments were limited by sensitivity and reproducibility of liquid chromatography-mass spectrometry and ELISA methods, respectively.

### Leptin protein accumulation and secretion is decreased by DHT treatments

**Figure 3:**
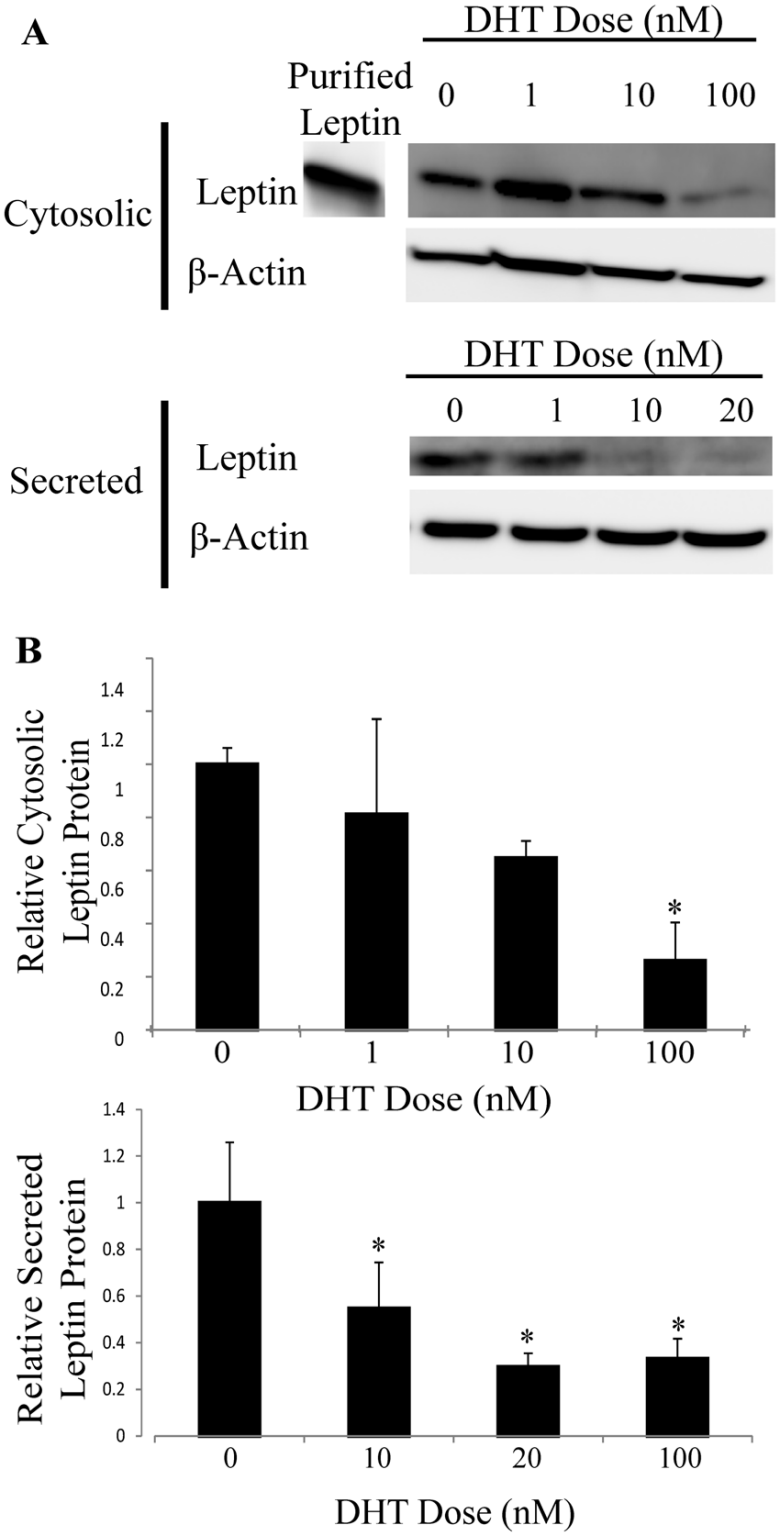
Leptin levels significantly decreased in both cell extracts and supernatants from cells treated with 1–100 nM DHT. Cells were treated with DHT for 24 hours after a 6 hour serum deprivation. (**A**) Cytosolic proteins were quantified in a cell extract and supernatants were collected for analysis with Western blots with a leptin-specific antibody. (**B**) Protein levels were normalized to actin and are reported relative to the untreated control with n ≥ 5 biological replicates; *Denotes p-value ≤ 0.05 when compared to control with 2-sample, independent Student’s t-tests. The blots in this image are cropped and full length images are shown in Supplemental Figs 3 and 4.

To determine if the changes in *Lep* transcripts were associated with changing levels of leptin protein upon steroid hormone treatment, we examined leptin protein levels by immunoblot. Five separate samples of protein (5 biological replicates) were used for this analysis, yielding two samples: A cell extract with proteins that were retained within cells (and accumulated in cell extracts) and secreted proteins in the media surrounding cells before their extraction were both examined by immunoblot. A representative immunoblot in Fig. 3A illustrates that when cells were treated with 10–100 nM DHT, significant decreases in leptin protein levels were evident in both cytoplasmic (*p* = 0.0001 at 100 nM) and secreted samples (*p* ≤ 0.02 for 10, 20, and 100 nM). The chemiluminescent signal was significantly decreased in response to DHT treatment in cytosolic samples to 30% of control values and in secreted leptin to 30–60% of controls (Fig. 3). These results suggest that changes in *Lep* transcript were paralleled by changes in leptin protein synthesis and secretion.

### 17β-Estradiol treatment increased *Lep* transcript accumulation

**Figure 4:**
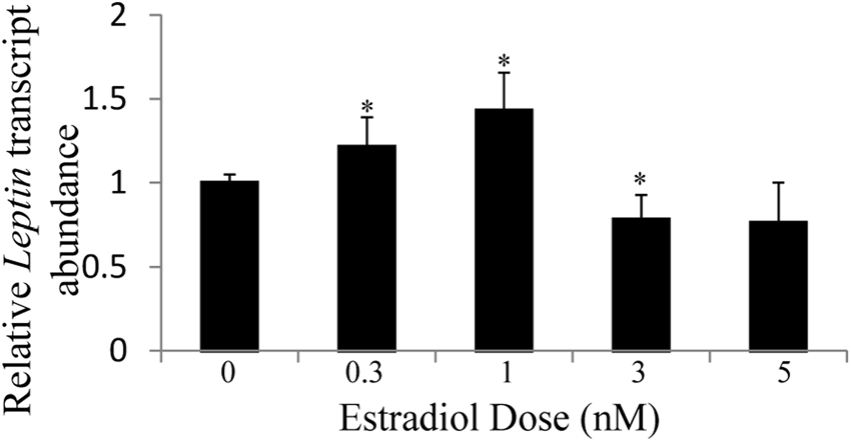
*Lep* transcript abundance increased at estradiol doses of 0.3 and 1 nM. 3T3-L1 adipocytes were treated for 24 hrs with estradiol doses ranging from 0.3–5 nM. Leptin transcripts were measured by qRT-PCR and normalized to an 18S rRNA control. The transcript abundance relative to untreated controls is reported as mean ± S.E.M of n ≥ 7 biological replicates; *Denotes p-value ≤ 0.05 when compared to the control with 2-sample, independent Student’s t-tests.

We also tested the secondary hypothesis that *Lep* transcripts are positively regulated by estradiol levels. *Lep* transcript abundance was measured after 24 hour treatments with 17β-estradiol at doses between 0.3 and 5 nM (Fig. 4). Statistically significant 1.2-fold and 1.4-fold increases in leptin transcript abundance relative to samples with no added hormone were observed at 0.3 and 1 nM (*p* ≤ 0.05) 17β-estradiol, respectively. These doses are close to physiological levels (0.1–0.3 nM) observed in premenopausal women during the early follicular phase of the menstrual cycle^53, 54^, but extreme levels as high as 5.5 nM have been reported during the mid-luteal phase^55^. The estradiol dose response curve was roughly bell shaped with doses higher than 1 nM leading to a decrease in transcript abundance (Fig. 4), consistent with other estradiol response curves using estradiol concentrations that are equal to or greater than the upper end of physiological levels^56, 57^. The elevated *Lep* transcripts at physiological doses suggest transcriptional controls that may lead to elevated leptin protein in females.

Although estradiol treatment increased *Lep* transcripts by a statistically significant 1.4-fold, we explored technical and biological reasons that might have limited the estrogen response. We explored the possibility that residual estradiol in the serum used to make the media could be elevating the level of *Lep* transcripts in the untreated controls, thereby minimizing the effects of treatment with exogenous estradiol. The effect of estradiol on *Lep* transcript abundance was compared in media prepared with regular serum or charcoal-striped serum. In the absence and presence of added estradiol, the *Lep* transcript abundance was not significantly different between the two types of sera (Supplemental Fig. 2), although the magnitude of the increase by 0.3 nM estradiol was greater in the charcoal-striped serum.

### Estrogen receptors are increased by 17β-estradiol treatment and ER antagonist block *Lep* transcript abundance changes

**Figure 5:**
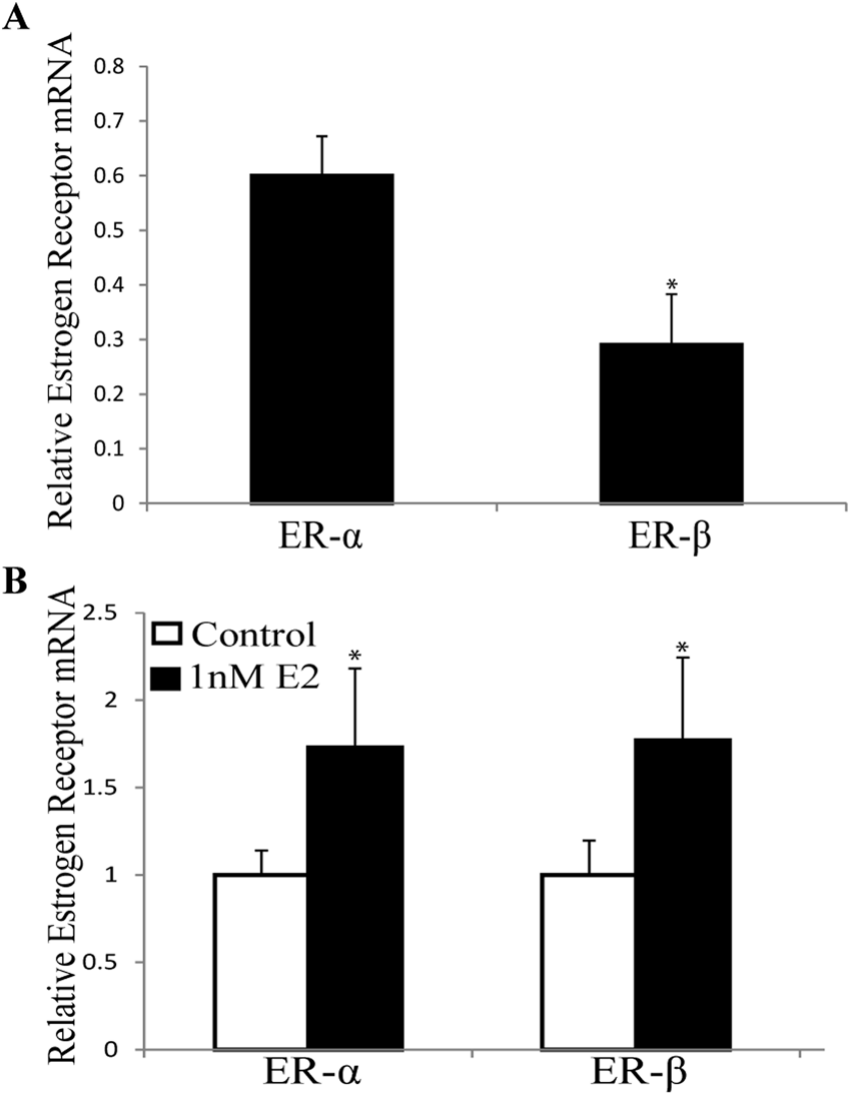
Transcripts encoding estrogen receptor (ER)-α and ER-β are estrogen-responsive in 3T3-L1 cells. Transcript abundance was determined by qRT-PCR and normalized to 18 S controls. (**A**) ER-α transcript abundance is greater than ER-β. *Denotes p-value ≤ 0.05 where levels of the receptor transcripts are compared. (**B**) When treated with 1 nM estradiol, the abundance of transcript of both receptors were increased. The values are normalized to untreated controls for each transcript to illustrate that both receptors have similar induction by estradiol. Mean ± S.E.M; n ≥ 3; *Denotes p-value ≤ 0.05 when compared to control with 2-sample, independent Student’s t-test.

We also verified that estrogen receptors were expressed in this cell type and that their transcript levels were modulated by estradiol treatment. Levels of the ERα and ERβ transcripts were quantified by qRT-PCR. The adipocytes expressed both receptors, with statistically significant higher levels of the α than the β isoform (*p* = 0.008) (Fig. 5A). Upon treatment with 1 nM estradiol, the abundance of transcripts for both the α and β receptors showed significant increases (1.7-fold change for both; *p* ≤ 0.05) (Fig. 5B), indicating that 3T3-L1 cells are estrogen-responsive.

**Figure 6:**
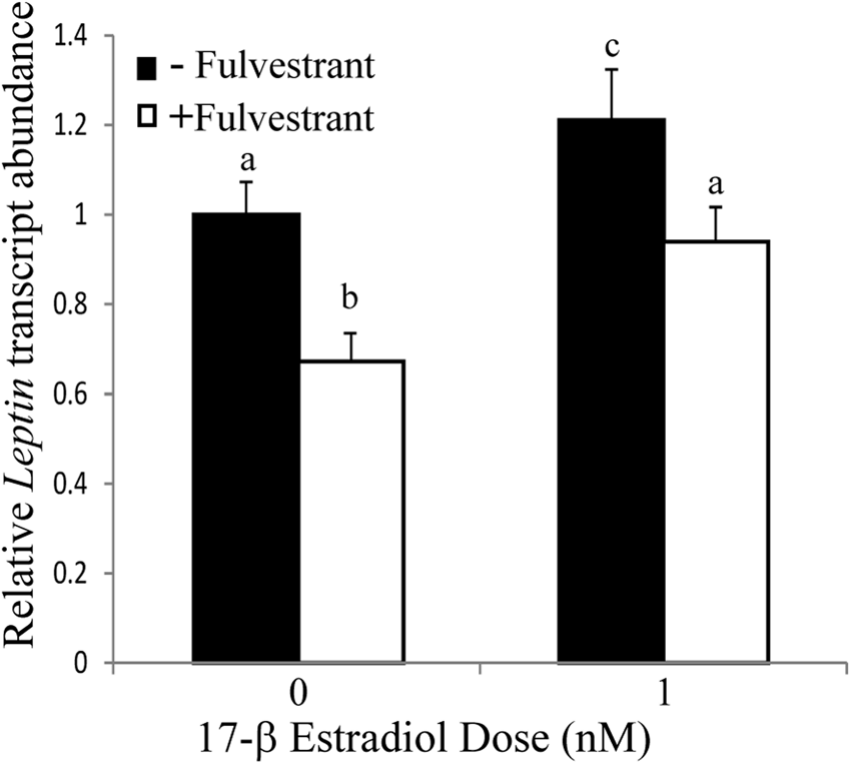
*Lep* transcript abundance decreased with fulvestrant treatment in the presence and absence of 1 nM estradiol. 3T3-L1 cells were treated for 24 hrs with estradiol, and cells were pretreated for 2 hrs with fulvestrant before incubation with estradiol. *Lep* transcript abundance was measured with qRT-PCR and normalized to an 18S rRNA control. Values are the mean ± S.E.M of n ≥ 5 biological replicates. Means with different letters are statistically different using two-tailed Mann-Whitney tests.

To provide additional insight into the regulation of leptin transcripts by estrogen receptor signaling, we used a well characterized inhibitor of both α and β estrogen receptors, fulvestrant^58^ and treated with this antagonist in the presence and absence of 17β-estradiol. A Kruskal-Wallis test examined the difference in transcript abundance between the different treatments, χ^2^(2) = 14.885, *p* = 0.002. The statistical significance between treatments was examined in pairwise Mann-Whitney comparisons. Treatment with 1 µM fulvestrant in the absence of exogenous steroid, led to a statistically significant decrease in *Lep* transcripts, down to 67% of the control value (*p* = 0.007) (Fig. 6), consistent with this inhibitor blocking endogenous levels of estradiol. Treatment with 1 nM estradiol increased *Lep* transcripts (*p* = 0.09), but the largest difference was a > 1.7-fold difference between estradiol and fulvestrant treatments (*p* ≤ 0.0008). The treatment with both inhibitor and estradiol returned *Lep* transcript abundance to levels not significantly different from untreated controls (*p* = 0.7) (Fig. 6), consistent with the competition between the binding of these two molecules to the estrogen receptor.

### Leptin protein secretion is increased by estradiol treatments

**Figure 7:**
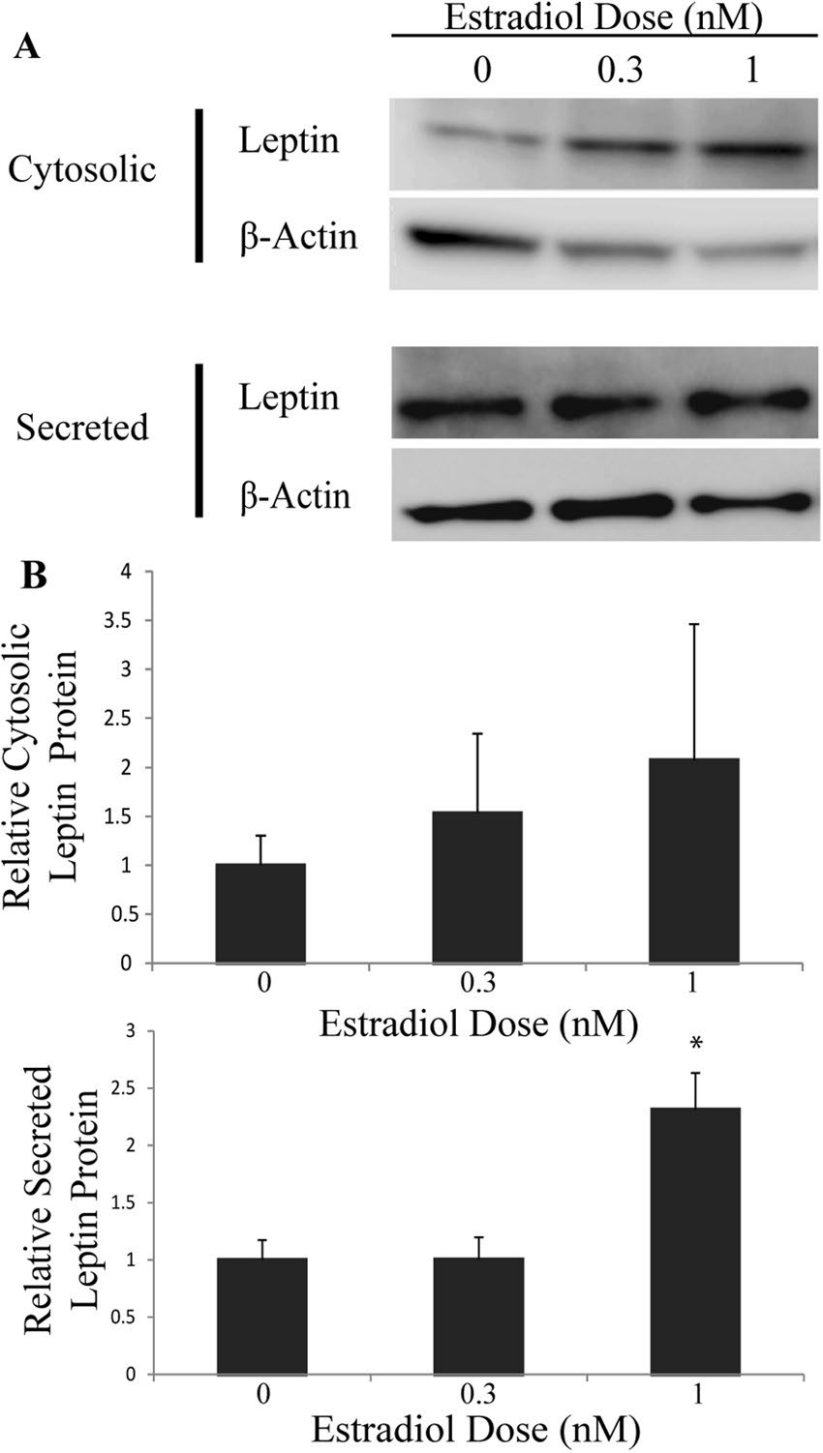
Secreted leptin significantly increased in cells treated with 1 nM estradiol. Cells were treated for 24 hours with estradiol after a 6 hour serum deprivation. (**A**) Cytosolic proteins were quantified in a cell extract and supernatants were collected for analysis with Western blots with a leptin-specific antibody. (**B**) Protein levels were normalized to actin and are reported relative to the untreated control with n ≥ 5 biological replicates; *Denotes p-value ≤ 0.05 when compared to control with 2-sample, independent Student’s t-tests. The blots in this image are cropped and full length images are shown in Supplemental Fig. 5.

Treatment with 1 nM estradiol led to a significant 2.3-fold increase (*p* = 0.003) in leptin protein detected by immunoblot of secreted protein samples compared to the control (Fig. 7). There was also a trend for increased leptin antibody signal with extracts from cells treated with estradiol, consistent with the blot in Fig. 7A, although the difference was not statistically significant. These effects of steroid hormones on leptin protein accumulation and secretion were more prominent in response to androgen treatment compared to estrogen treatments. As the animal studies that demonstrated sexual dimorphism in leptin levels in the bloodstream of males and females measure the summation of transcription, translation, and secretion of the leptin gene products, these studies demonstrate that estradiol and DHT affect the level of secreted leptin in 3T3-L1 adipocytes.

## Discussion

The sexual dimorphism in circulating levels of leptin, with higher levels in females, has been documented in multiple species^24, 30, 59, 60^. The timing of establishment of this dimorphism coincides with puberty^25^, suggesting that this dimorphism may be driven by steroid hormone changes that induce puberty, or the associated changes in abundance and distribution of body fat that occur during this transition^5, 22^. There are also sexual dimorphisms in fat stores with greater abundance of subcutaneous fat in females and visceral fat in males. These stores differ in amounts of leptin synthesis^30, 61^, suggesting that these two sexual dimorphisms may be connected^22^.

To test the possibility that the levels of sex steroid hormones modulate the synthesis and/or secretion of this important adipokine, independent of changes in fat depots, we used 3T3-L1 cells, a well-established pre-adipocyte cell line^43, 44^. Leptin synthesis occurs only after differentiation^62^ and this cell line has been used to demonstrate mechanisms that control leptin synthesis^21, 63^. We treated 3T3-L1 cells with 17β-estradiol and DHT to determine if *Lep* transcript abundance, leptin protein synthesis, and leptin protein secretion were altered by short-term treatments with sex steroid hormones, under conditions that did not alter fat cell differentiation.

DHT reduced *Lep* transcript abundance to 80% of untreated values, while estradiol increased levels to 140% of controls. As this study examined the basis of the sexual dimorphism in levels of circulating leptin, it was essential to examine the effect of steroid treatments on leptin secretion from 3T3-L1 cells. The effects of DHT and 17β-estradiol on leptin secretion are more profound than the effects on *Lep* transcript abundance or cytoplasmic leptin abundance. DHT reduced cytosolic and secreted leptin protein to ~30% of untreated controls, while estradiol increased secreted protein to 230% of untreated values. These results suggest the presence of post-transcriptional controls of leptin synthesis and secretion, for which this cell line is ideal for additional mechanistic studies.

We also examined the role of estrogen and androgen receptors in controlling *Lep* transcript abundance. We quantified the levels of estrogen receptors, finding a 2-fold higher level of ERα over ERβ, consistent with the more significant role of ERα in regulation of energy homeostasis^64, 65^ and with higher abundance of ERα than ERβ in female subcutaneous fat in some^66^, but not all studies^67^. The receptor antagonists, flutamide and fulvestrant, led to the opposite effects of DHT and estradiol, respectively, consistent with the canonical steroid hormone signaling pathways controlling synthesis of transcripts encoding leptin. These experiments were done in cells cultured in serum-free media for 30 hours before assays to deplete steroid hormone levels and allow inhibitors to out-compete remaining steroids. Finally, combined treatments with either steroid and its receptor antagonist reduced the opposite effects of both compounds, leading to values not different from treatment with either compound. This result is consistent with the flutamide and fulvestrant acting as competitive inhibitors, or antagonists, that block binding of these steroid hormones to their receptors.

Our results parallel and extend previous findings from primary cultures of rat adipocytes that reported a stimulatory effect of estradiol and inhibitory effect of DHT on *Lep* transcript abundance using non-quantitative PCR^30, 31^. In primary adipocyte cultures, isolated from rat subcutaneous fat deposits, estrogen treatments for 24 hours increased *Lep* mRNA levels by 140%, while treatment with DHT, reduced *Lep* transcripts to 70% of control values^30^. Effects elicited by DHT were ablated with the addition of the anti-androgen cyproterone acetate^30^. Another study, using human primary adipocytes that were isolated from female subcutaneous fat tissues, showed a pronounced increase in *Lep* mRNA (180–502%) and leptin secretion in response to estradiol treatments but no effect was observed in response to DHT treatment^31^. In contrast, similar adipocyte primary cultures from males showed a significant decrease in *Lep* transcript abundance and leptin secretion with DHT treatment but no effect of estradiol^31^. An additional study showed that the androgens DHT, dehydroepiandrosterone sulfate, and androstenedione decreased leptin secretion from omental adipose tissue extracted from human females, but not males^68^.

The regulation of *Lep* transcript accumulation by steroid hormones in 3T3-L1 cells was examined in two other studies^69, 70^. In one study, treatment with 10 µM 17β-estradiol led to a 22% increase in *Lep* transcript, while testosterone had no effect^70^, presumably since it is converted to both estradiol and DHT by aromatase and 5-alpha-reductase, respectively, which is present in these fat cells^70, 71^. In the second study focused only on estrogen effects, the authors were unable to detect increases in *Lep* or *ERα* transcripts upon treatment with 17β-estradiol, but they did find increases in *Lep* in response to treatment with an ERα agonist^69^. An ERα antagonist, 1,3-Bis(4-hydroxyphenyl)-4-methyl-5-[4-(2-piperidinylethoxy)phenol]-1H-pyrazole dihydrochloride, reduced *Lep* transcript abundance in an estradiol-reversible fashion. This study also differed in that they found higher levels of ERβ transcripts than ERα transcripts^69^, which contrasts with both our findings and another study^72^. Together, these agonist and antagonist treatments suggest that a higher ERα/ERβ ratio may be correlated with higher *Lep* transcript abundance and estradiol responses. Our ability to induce increases in *Lep* transcripts using a similar dose of estradiol may result from a difference in relative abundance of the ER receptor subtypes or the source of the 3T3-L1 cells. We obtained 3T3-L1 cells directly from the laboratory of Howard Green, (rather than ATCC) and used cells with a limited passage number. Our 3T3-L1 cells accumulated a 2-fold greater ratio of transcript abundance of ERα relative to ERβ, and our estradiol effects were abolished with the addition of the estrogen receptor inhibitor fulvestrant, which targets both ERα and ERβ^58^. The sexual dimorphism of leptin is likely linked to the fat depot specific expression of specific estrogen receptors as well as differences in sex steroid hormone levels between these depots. These results suggest that there may be fat store differences in estrogen-responsive leptin synthesis and secretion.

Another possible mechanism for elevated plasma leptin in females could be due to the sexual dimorphism of adipose tissue amounts and deposition, since leptin has been shown to be positively correlated with fat mass^59^. Because women tend to have more subcutaneous fat than men^22^, this could partially explain the differing amounts of leptin observed in men and women. However, studies that have corrected for fat mass between individuals have shown that females still have higher levels of serum leptin than males^73^. Our own study observed an increase in *Lep* transcript abundance with a 24 hr treatment of estradiol without an increase in lipid size or abundance as shown qualitatively by Oil Red O staining (Supplemental Fig. 1). Similarly, we detected no effect of DHT on fat deposition with this short treatment, which identified impaired lipid accumulation in 3T3-L1 cells in which DHT was present for 10 days during differentiation^45^. In addition, there have been studies showing a difference in leptin levels when sex steroid hormone levels have changed either with the onset of puberty, menopause, or the transition from one gender to another^28, 73–76^. For example, during menopause leptin levels drop concurrent with a decrease in estradiol levels, while fat mass usually increases^76^. These studies strongly suggest the varying ratios of sex steroid hormones present in each gender are important in maintaining the sexual dimorphism of leptin. One of the strengths of using 3T3-L1 cells is the ability to examine the effect of these steroid hormones in short term treatments where steroid effects on differentiation are not significant.

Understanding the sexual dimorphism in leptin levels is important as it relates to the clinical implications of obesity. How obesity affects an individual’s health seems to be dependent on many factors including the levels of sex steroid hormones and leptin. While leptin is an important signaling molecule involved in the regulation of appetite and metabolism under normal conditions, it has been documented that chronically elevated levels of leptin contribute to the systemic inflammation associated with obesity^77–79^. Chronically high levels of serum leptin have been implicated in the insulin-resistant state also associated with obesity and the metabolic syndrome^77, 80^. Since fat is an additional source of estrogen synthesis, it is possible that the higher levels of leptin during obesity are also due to an increase in fat-localized estrogens in addition to the larger fat mass^57, 81, 82^. Although the potential inflammatory effects of elevated leptin in females suggest a potential for concern, the majority of studies suggest that estrogens are protective.

Estrogen can be protective in rodents and women with high fat mass against visceral fat accumulation and cardiovascular disease^83, 84^. Women with moderately high leptin levels fared better than women with the lowest and highest amounts of plasma leptin, suggesting that at moderate fat levels leptin is protective^79^. A study in mice also showed that estrogen improved leptin sensitivity in obese female mice^84^. In addition, there is evidence that androgens can contribute to the risk for cardiovascular disease since their elevated levels associated with the accumulation of visceral fat leading to increased risk for obesity comorbidities such as cardiovascular disease and type 2 diabetes^85^. The increased levels of estrogen and leptin in women may be why women tend to be protected from the metabolic syndrome and cardiovascular disease compared to men^83^.

In conclusion, these results indicate that sex steroid hormones are involved in the regulation of leptin transcription and protein secretion in 3T3-L1 adipocytes. Elevated DHT decreased leptin transcript abundance, and levels of cytosolic and secreted leptin protein. Additionally, estradiol treatment resulted in increased levels of *Lep* transcripts and secreted protein levels suggesting a role for this steroid in sexual dimorphism. These results suggest the higher ratio of estrogen/androgen is in part responsible for higher leptin levels observed in females compared to males. Although the relationships among adipokines, obesity, and disease states is complex, these results provide evidence that sex steroid hormones are involved in the regulation of leptin transcription and protein synthesis and its sexual dimorphism.

## Electronic supplementary material

Supplemental data
