# Supplementary material for: Sex Steroid Hormones Regulate Leptin Transcript Accumulation and Protein Secretion in 3T3-L1 Cells
Source: Sci Rep. 2017 Aug 15;7:8232. doi: 10.1038/s41598-017-07473-5 (PMC5558017; doi:10.1038/s41598-017-07473-5)
Supplement: Supplementary file 1 — Supplemental data [file 41598_2017_7473_MOESM1_ESM.pdf]

Supplemental Materials for

Sex Steroid Hormones Regulate Leptin Transcript Accumulation and Protein Secretion in 3T3-L1 Cells

Mónica Z. Jenks, Heather E. Fairfield, Erik C. Johnson, Ron F. Morrison, Gloria K. Muday*

Supplemental Table 1. Primer sets used for quantitative real-time PCR

Target	Sequence
Leptin	F: 5’-ACCCCATTCTGAGTTTGTCC3’<br><br>R: 5’-TCCAGGTCATTGGCTATCTG-3’
18S	F: 5’-CCCTGCCCTTTGTACACACC-3’<br><br>R: 5’-CGATCCGAGGGCCTCACTA-3’
Estrogen Receptor alpha	F: 5’-AAGGCGGCATACGGAAAGA-3’<br><br>R: 5’-TCTGACGCTTGTGCTTCAACA-3’
Estrogen Receptor beta	F: 5’-CATCAGTAACAAGGGCATGGAA -3’<br><br>R: 5’-GTCGTACACCGGGACCACAT -3’

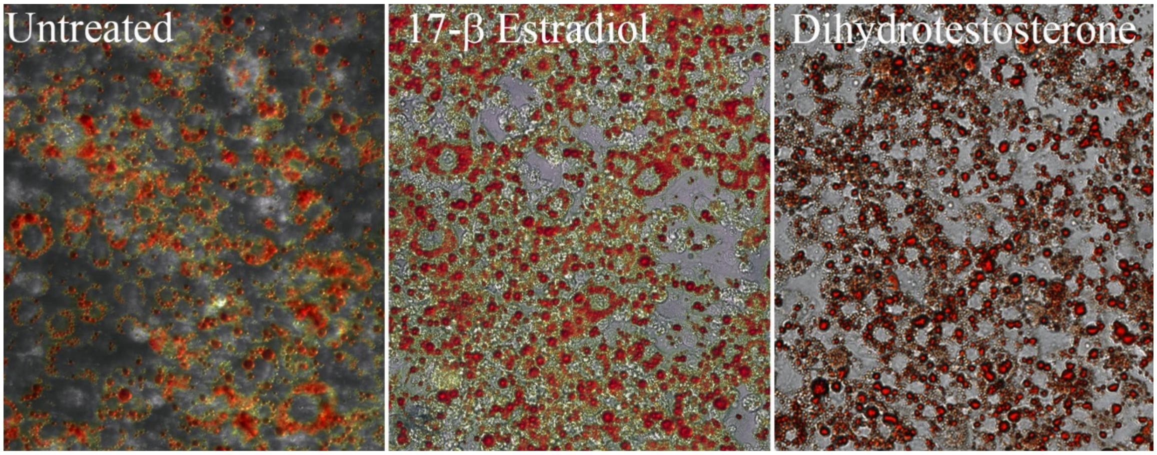

Supplemental Figure 1: Adipocytes were stained with Oil Red O dye 8 days after MDI treatment and imaged with an inverted light microscope and color camera. Samples were untreated control, 24 hr treatment with 1 nM estradiol, and 24 hr treatment with 20 nM DHT.

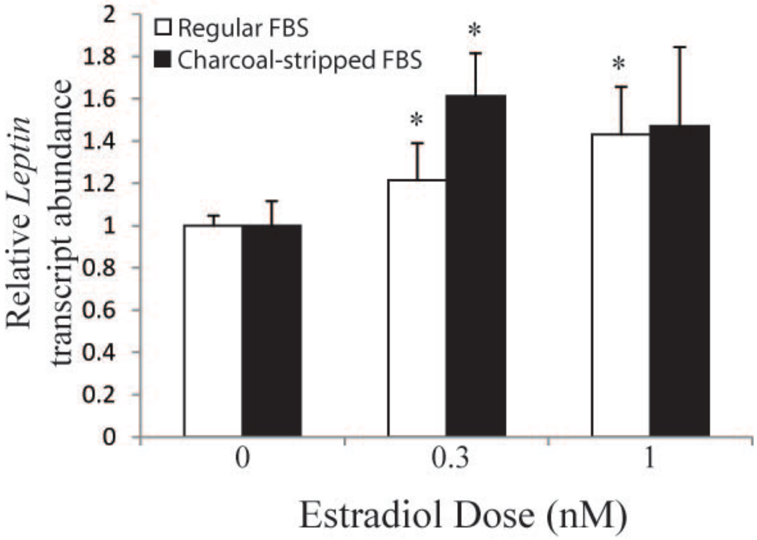

Supplemental Figure 2: 3T3-L1 cells were cultured in media with regular FBS until 5 days after differentiation when they were either kept in regular media or switched to media with charcoal-stripped FBS. Data are reported as mean ± S.E.M relative to the untreated control of $n \geq 3$ biological replicates. *denotes $p \leq 0.05$ when compared to the control with no estradiol within each media type. There was no significant difference in transcripts between serum types at each estradiol dose determined with 2-way ANOVA.

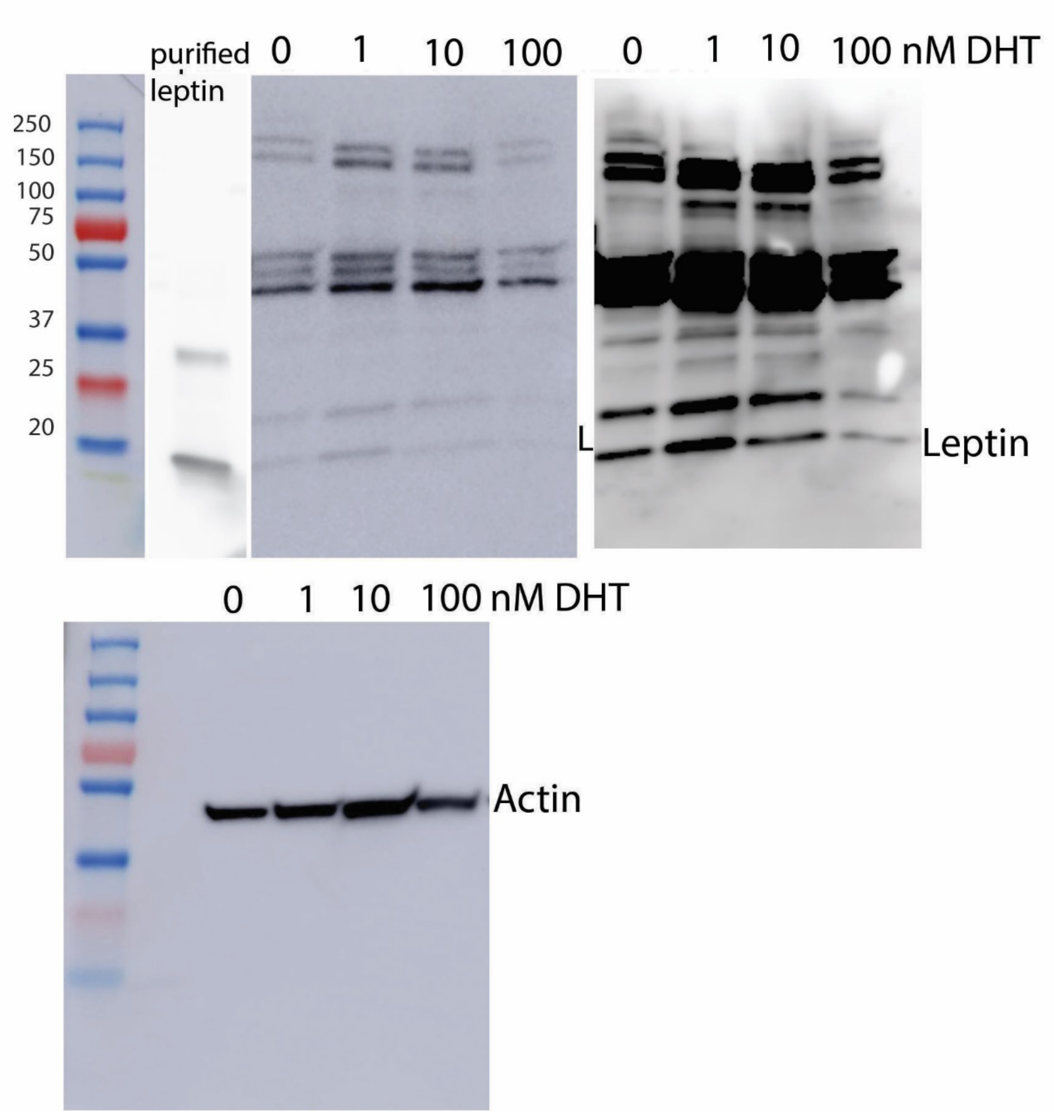

Supplemental Figure 3. Original uncropped blots for cytosolic samples treated with dihydrotestosterone shown in Figure 3. Purified mouse leptin (125 µg) of molecular weight 16kDa was used to demonstrate the leptin band recognized by this antibody, which recognizes several proteins. This standard was run on a separate gel from the 3T3-L1 samples, as when they were run together, the purified leptin titrated out the antibody resulting in insufficient signal from the 3T3-L1 samples to complete a quantitative analysis. The immunoblot of 3T3-L1 cytosolic samples was imaged with two different exposure times. The reactive band with molecular weight of leptin is noted. The actin standards used for normalization are shown in the lower blot.

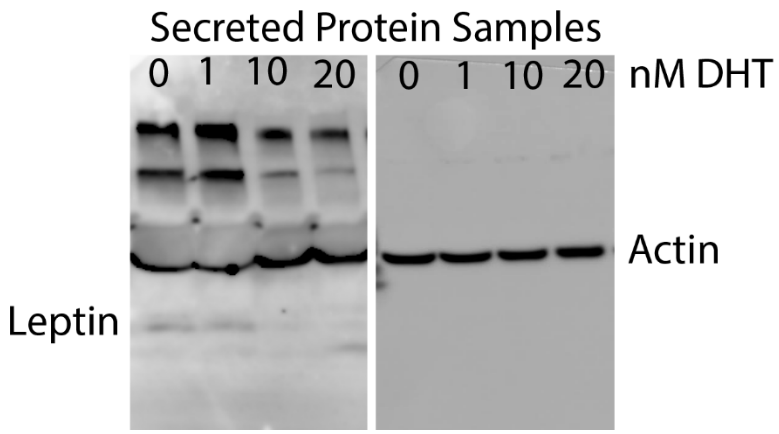

Supplemental Figure 4. Original uncropped blots for secreted dihydrotestosterone-treated protein samples from Figure 3. Purified mouse leptin (125 µg) of molecular weight of 16kDA was used to demonstrate the leptin band recognized by this antibody, which recognizes several proteins. The reactive band with the molecular weight of leptin is noted. The actin bands used for normalization are shown on the panel on the right.

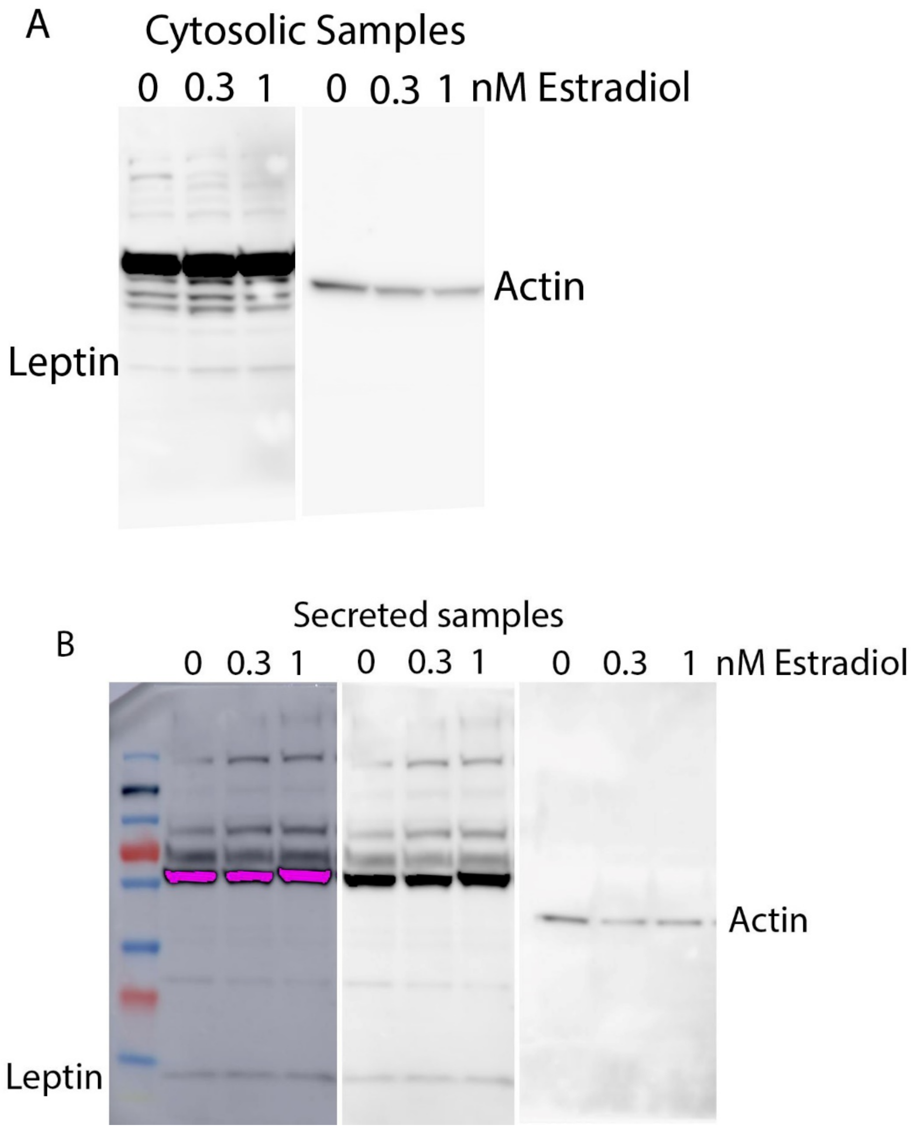

Supplemental Figure 5. Original uncropped blots for secreted 17β-estradiol treated protein samples from Figure 7. (A) The cytosolic samples examined by immunoblot with leptin antibody on the left and actin antibody on the right are compared, with the cross-reactive proteins of appropriate size indicated. (B) The secreted samples with the same leptin immunoblot shown with two outputs from the imager, either in a jpg file with the blot overlaid on top of the molecule weight standards (with the saturated samples shown in pink) or with a higher resolution tif file to its right. The reactive band with the molecular weight of leptin is noted. The actin standards used for normalization are shown in the panel on the right, and the band is also labeled.
